# Supplementary material for: Archetypal analysis of COVID-19 in Montana, USA, March 13, 2020 to April 26, 2022
Source: PLoS One. 2024 Jan 3;19(1):e0283265. doi: 10.1371/journal.pone.0283265 (PMC10763954; doi:10.1371/journal.pone.0283265)
Supplement: S1 File — (PDF) [file pone.0283265.s001.pdf]

Supporting information

The Matlab code used to produce these results can be found on github: Em878/PLOS-Arch. The efficient algorithm for computing the Principal Convex Hull (PCHA), was created by Morten Mørup. The code was written for Matlab by J.C. Thørgersen. Mørup's Principal Convex Hull software can be downloaded at `https://mortenmorup.dk/wp_updated/contact/`. We reproduce text versions of the code here, for completeness.

```
%% PCHA.m
function [XC,S,C,SSE,varexpl]=PCHA(X,noc,I,U,delta,varargin)
% Principal Convex Hull Analysis (PCHA) / Archetypal Analysis
%
% Written by Morten Mørup
%
% Usage:
%   [XC,S,C,SSE,varexpl]=PCHA(X,noc,W,I,U,delta,varargin)
%
%   Solves the following PCH/AA problem
%   \|X(:,U)-X(:,I)CS\|_F^2 s.t. |s_j|_1=1, 1-delta<=|c_j|_1<=1+delta,
%   S>=0 and C>=0
%
%
% Input:
% X             data array (Missing entries set to zero or NaN)
% noc           number of components
% I             Entries of X to use for dictionary in C (default: I=1:size(X,2))

% U             Entries of X to model in S              (default: U=1:size(X,2))
% delta         relaxation of C, i.e. 1-delta<=|C_j|_1<=1+delta (default: delta=0, i.e. |C_j|_1=1)
%
% opts.         Struct containing:
%       C            initial solution (optional) (see also output)
%       S            initial solution (optional) (see also output)
%       maxiter      maximum number of iterations (default: 500 iterations)
%       conv_crit    The convergence criteria (default: 10^-6 relative change in SSE)
%
% Output:
% XC            I x noc feature matrix (i.e. XC=X(:,I)*C forming the archetypes)
% S             noc x length(U) matrix, S>=0 |S_j|_1=1
% C             length(I) x noc matrix, C>=0 1-delta<=|C_j|_1<=1+delta
% SSE           Sum of Squares Error
% varexpl       Percent variation explained by the model
%
% Copyright (C) Morten Mørup and Technical University of Denmark, 2010
```

```
warning('off','MATLAB:dispatcher:InexactMatch')
if nargin>=6, opts = varargin{1}; else opts = struct; end
conv_crit=mgetopt(opts,'conv_crit',10^-6);
maxiter=mgetopt(opts,'maxiter',500);

if nargin<5
    delta=0;
end
if nargin<4
    U=1:size(X,2);
end
if nargin<3
    I=1:size(X,2);
end

SST=sum(sum(X(:,U).*X(:,U)));

% Initilize C
if isfield(opts,'C')
    C = opts.C;
else
   % Initialize by furthest sum
   i=FurthestSum(X(:,I),noc,ceil(length(I)*rand));
   C=sparse(i,1:noc,ones(1,noc),length(I),noc);
end
XC=X(:,I)*C;

muS=1;
muC=1;
mualpha=1;

% Initilize S
if isfield(opts,'S')
    S=opts.S;

    CtXtXC=XC'*XC;
    XSt=X(:,U)*S';
    SSt=S*S';
    SSE=SST-2*sum(sum(XC.*XSt))+sum(sum(CtXtXC.*SSt));


 else
```

```
    XCtX=XC'*X(:,U);
    CtXtXC=XC'*XC;
    S=-log(rand(noc,length(U)));
    S=S./(ones(noc,1)*sum(S));
    SSt=S*S';
    SSE=SST-2*sum(sum(XCtX.*S))+sum(sum(CtXtXC.*SSt));
    [S,SSE,muS,SSt]=Supdate(S,XCtX,CtXtXC,muS,SST,SSE,25);
end

% Set PCHA parameters
iter=0;
dSSE=inf;
t1=cputime;
varexpl=(SST-SSE)/SST;

% Display algorithm profile
%disp([' '])
%disp(['Principal Convex Hull Analysis / Archetypal Analysis'])
%disp(['A ' num2str(noc) ' component model will be fitted']);
%disp(['To stop algorithm press control C'])
%disp([' ']);
%dheader = sprintf('%12s | %12s | %12s | %12s | %12s | %12s | %12s | %12s','Iteration',
'Expl. var.','Cost func.','Delta SSEf.','muC','mualpha','muS',' 
%dline = sprintf('-------------+--------------+--------------+--------------+--------------+--------------+---------
-----+--------------+')

told=t1;
while abs(dSSE)>=conv_crit*abs(SSE) && iter<maxiter && varexpl<0.9999
 %   if mod(iter,100)==0
   %      disp(dline); disp(dheader); disp(dline);
   % end
    told=t1;
    iter=iter+1;
    SSE_old=SSE;

    % C (and alpha) update
    XSt=X(:,U)*S';
    [C,SSE,muC,mualpha,CtXtXC,XC]=Cupdate(X(:,I),XSt,XC,SSt,C,delta,muC,mualpha,SST,SSE,10);

    % S update
    XCtX=XC'*X(:,U);
    [S,SSE,muS,SSt]=Supdate(S,XCtX,CtXtXC,muS,SST,SSE,10);

    % Evaluate and display iteration
    dSSE=SSE_old-SSE;
    t1=cputime;
    if rem(iter,1)==0
        pause(0.000001);
        varexpl=(SST-SSE)/SST;
```

```
        % fprintf('%12.0f | %12.4f | %12.4e | %12.4e | %12.4e | %12.4e | %12.4e | %12.4f \n',iter,
        varexpl,SSE,dSSE/abs(SSE),muC,mualpha,muS,t1-told);
    end
end

% display final iteration
varexpl=(SST-SSE)/SST;
%disp(dline);
%disp(dline);
%fprintf('%12.0f | %12.4f | %12.4e | %12.4e | %12.4e | %12.4e | %12.4e | %12.4f \n',iter,
varexpl,SSE,dSSE/abs(SSE),muC,mualpha,muS,t1-told);

% sort components according to importance
[val,ind]=sort(sum(S,2),'descend');
S=S(ind,:);
C=C(:,ind);
XC=XC(:,ind);

% ------------------------------------------------------------------------
% Parser for optional arguments
function var = mgetopt(opts, varname, default, varargin)
if isfield(opts, varname)
    var = getfield(opts, varname);
else
    var = default;
end
for narg = 1:2:nargin-4
    cmd = varargin{narg};
    arg = varargin{narg+1};
    switch cmd
        case 'instrset',
            if ~any(strcmp(arg, var))
                fprintf(['Wrong argument %s = ''%s'' - ', ...
                    'Using default : %s = ''%s''\n'], ...
                    varname, var, varname, default);
                var = default;
            end
        otherwise,
            error('Wrong option: %s.', cmd);
    end
end


% ------------------------------------------------------------------------
function [S,SSE,muS,SSt]=Supdate(S,XCtX,CtXtXC,muS,SST,SSE,niter)

    [noc,J]=size(S);
    e=ones(noc,1);
    for k=1:niter
        SSE_old=SSE;
        g=(CtXtXC*S-XCtX)/(SST/J);
        g=g-e*sum(g.*S);
        stop=0;
        Sold=S;
```

```
        while ~stop
            S=Sold-g*muS;
            S(S<0)=0;
            S=S./(e*sum(S));
            SSt=S*S';
            SSE=SST-2*sum(sum(XCtX.*S))+sum(sum(CtXtXC.*SSt));
            if SSE<=SSE_old*(1+1e-9)
                muS=muS*1.2;
                stop=1;
            else
                muS=muS/2;
            end
        end
    end

%---------------------------------------------------------------------
function [C,SSE,muC,mualpha,CtXtXC,XC]=Cupdate(X,XSt,XC,SSt,C,delta,muC,mualpha,SST,SSE,niter)

    [J,noc]=size(C);
    if nargin<12
        niter=1;
    end
    if delta~=0
        alphaC=sum(C);
        C=C*diag(1./alphaC);
    end
    e=ones(J,1);
    XtXSt=X'*XSt;
    for k=1:niter

        % Update C
        SSE_old=SSE;
        g=(X'*(XC*SSt)-XtXSt)/SST;
        if delta~=0
            g=g*diag(alphaC);
        end
        g=g-e*sum(g.*C);
        stop=0;
        Cold=C;
        while ~stop
            C=Cold-muC*g;
            C(C<0)=0;

            nC=sum(C)+eps;
            C=C*sparse(1:noc,1:noc,1./nC);
            if delta~=0
                Ct=sparse(C*diag(alphaC));
            else
                Ct=sparse(C);
            end
            XC=X*Ct;
            CtXtXC=XC'*XC;
```

```
            SSE=SST-2*sum(sum(XC.*XSt))+sum(sum(CtXtXC.*SSt));
            if SSE<=SSE_old*(1+1e-9)
                muC=muC*1.2;
                stop=1;
            else
                muC=muC/2;
            end
        end

        % Update alphaC
        SSE_old=SSE;
        if delta~=0
            g=(diag(CtXtXC*SSt)'./alphaC-sum(C.*XtXSt))/(SST*J);
            stop=0;
            alphaCold=alphaC;
            while ~stop
                alphaC=alphaCold-mualpha*g;
                alphaC(alphaC<1-delta)=1-delta;
                alphaC(alphaC>1+delta)=1+delta;
                XCt=XC*diag(alphaC./alphaCold);
                CtXtXC=XCt'*XCt;
                SSE=SST-2*sum(sum(XCt.*XSt))+sum(sum(CtXtXC.*SSt));
                if SSE<=SSE_old*(1+1e-9)
                    mualpha=mualpha*1.2;
                    stop=1;
                    XC=XCt;
                else
                    mualpha=mualpha/2;
                end
            end
        end
    end
    if delta~=0
        C=C*diag(alphaC);
    end


%% make_arch.m
noc=3; % Number of archetypes
U=1:size(X,2); % Entries in X used that is modelled by the AA model
I=1:size(X,2); % Entries in X used to define archetypes
% if two expensive to useall entries for I find N relevant observations by
% the following procedure:
% N=100;
% I=FurthestSum(X,N,ceil(rand*size(X,2)));

delta=0;
opts.maxiter=5000;
opts.conv_crit=1e-6;
clear XC
% Use PCHA.m
[XC,S,C,SSE,varexpl]=PCHA(X,noc,I,U,delta,opts);
```

```
XX=XC*S;


%%make_data.m
M=size(dat);
N=M(1);
I=ones(N,1);   %Identity matrix dim of number of samples
population=I*pop';   %pop is number of counties by 1, so population is number of samples by number
                     of counties
 norm=dat./opulation*1000;     %can now do an elementwise division of dat by pop

%%countybycountyMI
%computes MI for all pairs of districts.  MI matrix will be symmetric
clear MI
%number of bins
Nx=30;
Ny=30;
N=676;  %number of weeks
M=56;  %number of counties
MI=zeros(M,M);
H=zeros(M,M);
num=linspace(1,M,M);
for k=1:M
    for l=1:M

         C1=struncdat(:,k);
         C2=struncdat(:,l);

        E=zeros(Nx,1);
        EY=zeros(Nx,1);
        EXY=zeros(Nx,1);

        %make frequency tables

        C1dist=hist(C1,Ny)/N;
        C2dist=hist(C2,Nx)/N;

        v=[C1 C2]; %samples to be binned up
        PP=hist3(v, [Nx,Ny]);   %make a 2D histogram of both, with given bin sizes
        Pdist=PP/(N);   %normalize it

        %use entropy formulas for pdfs
        %compute H(X,Y) joint entropy
        sumH=0;
        for j=1:Nx
            sum1=0;
            for i=1:Ny
                if Pdist(j,i) ~= 0
                    sum1=sum1+Pdist(j,i)*log2(Pdist(j,i));
                end
            end
            sumH=sumH+sum1;
```

```
        end

        sum2=0;
        %compute H for D2 (X)
        for i=1:Nx
            if C2dist(i) ~= 0
                sum2=sum2+C2dist(i)*log2(C2dist(i));
            end
        end

        sum3=0;
        %compute H for D1 (Y)
        for i=1:Ny
            if C1dist(i) ~= 0
                sum3=sum3+C1dist(i)*log2(C1dist(i));
            end
        end

        HX=-sum2;
        HY=-sum3;
        HXY=-sumH;

        %MI=HY+HX-HXY
        MI(k,l)=(HY+HX-HXY)/(HX+HY)*2;
        H(k,l)=HXY;

    end
end
tot=sum(MI);  %sum up MI with all other couties for each county

MItot=[num' tot'];
sortedMItot=bubblevecsort(MItot);
figure
plot(sortedMItot(:,2))
```
